# Epimedium for Osteoporosis Based on Western and Eastern Medicine: An Updated Systematic Review and Meta-Analysis

**DOI:** 10.3389/fphar.2022.782096

**Published:** 2022-03-31

**Authors:** Shihua Shi, Fei Wang, Yong Huang, Bonan Chen, Caixia Pei, Demei Huang, Xiaomin Wang, Yilan Wang, Shuo Kou, Weihao Li, Tianhong Ma, Yongcan Wu, Zhenxing Wang

**Affiliations:** ^1^ Hospital of Chengdu University of Traditional Chinese Medicine, Chengdu, China; ^2^ Department of Anatomical and Cellular Pathology, State Key Laboratory of Translational Oncology, Prince of Wales Hospital, The Chinese University of Hong Kong, Hong Kong, Hong Kong,SAR, China; ^3^ School of Public Health, Zhejiang Chinese Medical University, Hangzhou, China; ^4^ Cardiology Division, West China Hospital, Sichuan University, Chengdu, China; ^5^ College of Traditional Chinese Medicine, Chongqing Medical University, Chongqing, China; ^6^ Chongqing Key Laboratory of Traditional Chinese Medicine for Prevention and Cure of Metabolic Diseases, Chongqing, China

**Keywords:** epimedium, osteoporosis, meta-analysis, bone density, Visual Analog Scale, alkaline phosphatase

## Abstract

**Background:** The efficacy of conventional pharmacotherapy on osteoporosis was limited and accompanied with serious side effects. Epimedium might have the potential to be developed as agents to treat osteoporosis. The present systematic review and meta-analysis integrating Western medicine and Eastern medicine (“WE” medicine) was to evaluate the efficacy of Epimedium on osteoporosis.

**Methods:** Eleven electronic databases were searched to identify the randomized controlled trials (RCTs) comparing Epimedium as an adjunctive or alternative versus conventional pharmacotherapy during osteoporosis. Bone mineral density (BMD), effective rate, and Visual Analog Scale (VAS) were measured as primary outcomes. The secondary outcomes were pain relief time, bone metabolic markers, and adverse events. Research quality evaluation was conducted according to the modified Jadad scale. Review Manager 5.4 was utilized to perform analyses, and the data were pooled using a random-effect or fixed-effect model to calculate the weighted mean difference (WMD), standardized mean difference (SMD), risk ratio (RR), and 95% confidence intervals (CI).

**Results:** Twelve RCTs recruiting 1,017 patients were eligible. Overall, it was possible to verify that, in the Epimedium plus conventional pharmacotherapy group, BMD was significantly improved (*p* = 0.03), effective rate was significantly improved (*p* = 0.0001), and VAS was significantly decreased (*p* = 0.01) over those in control group. When compared to conventional pharmacotherapy, Epimedium used alone improved BMD (*p* = 0.009) and effective rate (*p* < 0.0001). VAS was lower (*p* < 0.00001), and the level of alkaline phosphatase (ALP) was significantly decreased (*p* = 0.01) in patients taking Epimedium alone compared with those given conventional pharmacotherapy. Results of subgroup analyses yielded that the recommended duration of Epimedium as an adjuvant was >3 months (*p* = 0.03), the recommended duration of Epimedium as an alternative was ≤3 months (*p* = 0.002), and Epimedium decoction brought more benefits (SMD = 2.33 [1.92, 2.75]) compared with other dosage forms. No significant publication bias was identified based on statistical tests (t = 0.81, *p* = 0.440).

**Conclusions:** Epimedium may improve BMD and effective rate and relieve pain as an adjuvant or alternative; Epimedium as an alternative might regulate bone metabolism, especially ALP, with satisfying clinical efficacy during osteoporosis. More rigorous RCTs are warranted to confirm these results.

## Introduction

Osteoporosis is a chronic bone lytic disease attributed to imbalance between bone ossification and bone resorption characterized by decreased bone mass, altered bone micro-architecture, and increased bone fragility (Li et al., 2019). Bone loss gradually and asymptomatically occurs with aging and is related to high fracture risk, with an accident of osteoporotic fracture every 3 s, contributing to disability, death, and worldwide healthcare expenditure (Tomasevic-Todorovic et al., 2018). With a prevalence of 200–300 million patients worldwide (Mellado-Valero et al., 2010; Sözen et al., 2017), osteoporosis has become a major public health concern. However, the treatment of osteoporosis remains a challenge around the world (Li et al., 2019), since the clinic use of conventional pharmacotherapy including estrogen therapy, bisphosphonates, and anabolic agents is usually accompanied with various serious side effects including the exacerbation of hot flushes, increased risk of venous thrombo-embolism, atrial fibrillation, atypical fractures, and osteonecrosis of the jaw (Indran et al., 2016). Given the complications and limited efficacy of currently available treatments (Foidart et al., 2007), developing new therapeutic approaches for the prevention and intervention of osteoporosis like phytomedicine may be promising.

Improving the use of other medicine (accessary medicine) is one of the scopes of medicine needed today. “WE” medicine is a concept proposed by pharmacology professor Yung-Chi Cheng at Yale University, which is a new trend of future medicine, mining ancient botanical drugs for cutting-edge therapies. “WE” medicine is a melding of Western medicine and Eastern medicine (Shi et al., 2021). Western medicine is focused on microscopic and single-disease targets, and Eastern medicine can be exemplified by traditional Chinese therapies that are based on a holistic medical system for diagnosis, prevention, and treatment of diseases for thousands of years (Cheng and Belli, 2020; Shi et al., 2021). Professor Cheng believes that multiple targets should be focused on, and polychemical medicine instead of one chemical medicine with system biology approach should be considered in the new paradigm for future medicine. Chinese botanical drugs have many chemicals which could target on multiple sites or act on a single site additively or synergistically through direct or in direct interaction. Chinese medicine takes a holistic approach and is an early form of “system biology” based “integrated medicine.” Phytotherapy has been safely used for thousands of years, attracting great attention worldwide (Liu et al., 2018). Botanical drugs have played a vital role in treating many diseases, even cancer (Lam et al., 2010) and epidemic disease including coronavirus disease 2019, which is considered as a valuable but underestimated part of health care, and the World Health Organization (WHO) has set out a plan for Traditional and Chinese Medicine development (WHO Traditional Medicine Strategy 2014–2023) (Indran et al., 2016; Xiong et al., 2020). The logical use of botanical drugs may affect the course of conditions, syndromes, and diseases to benefit the health of an individual. Our research focused on illustrating “WE” medicine by studying the efficacy of one traditional Chinese therapy through evidence-based medicine, which integrates Western medicine and Eastern medicine.

Epimedium, derived from the dried leaf of the *Epimedium wushanense* T. S. Ying, *Epimedium brevicornu* Maxim, *Epimedium sagittatum* (Siebold & Zucc.) Maxim., *Epimedium pubescens* Maxim., or *Epimedium koreanum* Nakai, has been used broadly in East Asia (e.g., China, Japan, and Korea) (Yu et al., 2013). Epimedium could be found in tea, condiments, and other food items in China, since it is rather safe with few side effects, and is defined as homology of medicine and food by National Health Commission of the People’s Republic of China (China, 2002; Sun et al., 2020). The popular usages of Epimedium include treating sexual dysfunction (impotence, prospermia, spermatorrhoea), cardiovascular and cerebrovascular diseases, menstrual irregularity, menopause syndrome, asthma, chronic tracheitis, and chronic nephritis, and in modulating immunological function (Ma et al., 2011; Dietz et al., 2016). In traditional Chinese medicine (TCM), Epimedium (horny goat weed), with the function of nourishing the kidney and reinforcing the Yang, has been widely used as an aphrodisiac, antirheumatic, or a tonic to prevent disease and strengthen the body. Multiple active compounds of Epimedium, having antioxidation, anti-hypoxia, anti-fatigue, anti-tumor, anti-aging, anti-inflammatory, anti-virus, anti-bacterial, anti-atherosclerosis, anti-depressant, and anti-hepatotoxic effects, which have been illustrated in *in vivo* and *in vitro* experiments and clinical practice (Ma et al., 2011), contribute to the TCM function of reinforcing the Kidney Yang, based on “WE” medicine.

Epimedium has also been used in treating bone diseases based on empirical TCM knowledge. It was believed that Epimedium could modulate bone health in positive ways, and may be beneficial for tendon health (Meng et al., 2005). The views of potential targets of Epimedium on osteoporosis have developed further recently. Epimedium works by taking advantage of multiple chemicals, and recent studies have reported striking pharmacological activity on cells and tissues of the muscular skeletal system of Epimedium extracts like icariin, lignins, flavonoids, genistein, daidzein, and many other compounds, which can benefit bone health via a variety of signaling pathways, including RANKL/RANK, ROS, or estrogen signaling pathways (Ma et al., 2011), and elicit osteogenic effects through targeting mesenchymal stem cells, osteoblast and osteoclast differentiation, proliferation, or function, thus improving bone health (Indran et al., 2016). A recent study based on high-throughput metabolomics method for discovering metabolic biomarkers and pathways demonstrated that the pharmacological effect and action mechanism of ethanol extract of Epimedium on osteoporosis rat model were related with glycerolphospholipid and sarachidonic acid metabolism (Zhao et al., 2020). According to “WE” medicine, Epimedium has the potential to be developed as an agent with other drugs to prevent or delay the onset and development of osteoporosis.

The efficacy of Epimedium-containing herbal formula for osteoporosis has been suggested by several reviews (Zhai et al., 2013; Wang et al., 2016). However, the findings regarding the effect of Epimedium as a single botanical drug are not fully conclusive. For instance, on the one hand, Wang demonstrated that Epimedium has no advantage on improving Ca^2+^ (Wang, 2002); on the other hand, one study illustrated that Epimedium significantly improved Ca^2+^ more than the control group (Zhao, 2003). The effect of Epimedium as single botanical drug alone or combined with conventional pharmaceutical treatment on osteoporosis remains poorly understood clinically and has not been evaluated systematically, though not only eastern medicine, exemplified by “Shennong’s Classic of Materia Medica,” a famous Chinese herbal medicine classic books dated back to around 220AD, but also western medicine, exemplified by modern research including cellular and animal studies (Indran et al., 2016) has shown that Epimedium was effective and non-toxic. Admittedly, the therapeutic uses of a botanical drug were controversial sometimes, and the relevant studies were not exhaustive, especially when the botanical drug was used as an adjuvant. Some experts even doubted that the efficacy botanical drugs brought in combination therapy with conventional pharmacotherapy was a placebo effect. To identify the effect of Epimedium as an alternative or adjuvant based on current clinical studies, we conducted this systematic review and meta-analysis, studying Epimedium as a single botanical drug for osteoporosis compared with conventional pharmacotherapy. To avoid the suspect effect and resolve any potential controversy, we investigated Epimedium as a single botanical drug for osteoporosis from comprehensive aspects including not only subjective indicators like pain intensity using a Visual Analog Scale (VAS), but objective indicators including bone mineral density (BMD) and bone metabolic markers, since we were aimed at showing a balanced, comprehensive, and critical view of the literature in the field. The present study also tried to figure out the mode of action by botanical drug and the potential mechanism of Epimedium acting in osteoporosis by evaluating the biochemical markers of bone metabolism.

## Methods

### Study Registration

We designed this study following the Preferred Reported Items for Systematic Review and Meta-analysis 2020 statement (the PRISMA 2020) (Page et al., 2021). An additional file shows this in more detail (Supplementary Table S1). The study protocol was registered with OSF (DOI 10.17605/OSF.IO/GHFJB).

### Search Strategies

The search strategy aimed to identify randomized controlled trials (RCTs) comparing Epimedium alone or combined with conventional pharmaceutical treatment on osteoporosis. Systematic literature searches in Web of Science, WorldCat, Cochrane Library, EMBASE, Science Direct, Google Scholar, PubMed, SinoMed, Chongqing VIP Chinese Science and Technology Periodical Database, China National Knowledge Infrastructure (CNKI), and Wanfang Data were conducted. All the electronic databases were searched from the earliest available date to March 30, 2021 (updated September 21, 2021). In order to collect a more comprehensive data, a combination of the following English terms was used in the database searches: (“Epimedium” or “Epimediums” or “Epimedium sagittatum” or “Epimedium sagittatums” or “sagittatum, Epimedium” or “sagittatums, Epimedium” or “Epimedium grandiflorum” or “Epimedium grandiflorums” or “grandiflorum, Epimedium” or “grandiflorums, Epimedium” or “Epimedii Folium”) and (“Osteoporosis” or “Osteoporosis, Senile” or “Osteoporoses, Senile” or “Senile Osteoporoses” or “Senile Osteoporosis” or “Osteoporosis, Age Related” or “Bone Loss, Age-Related” or “Age-Related Bone Loss” or “Age-Related Bone Losses” or “Bone Loss, Age Related” or “Bone Losses, Age-Related” or “Age-Related Osteoporosis” or “Age Related Osteoporosis” or “Age-Related Osteoporoses” or “Osteoporoses, Age-Related”) and “randomized controlled trial.” For the Chinese databases, the following keywords were used in combination: (“Yinyanghuo” or “Xianlingpi”) and “Osteoporosis” and “randomized controlled trial.” Furthermore, reviews and the reference lists of all the related articles were screened to check for potential eligible RCTs. Search strategies for all databases could be found in the supplementary material (Supplementary Table S2). Primary outcomes included BMD which was measured by dual-energy X-ray bone density analyzer before and after the treatment, clinical efficacy (effective: the clinical symptoms such as pain and fatigue were relieved, BMD increased than that before treatment, and functional activities significantly improved [Liang et al., 2021]), and pain intensity using a VAS. Secondary outcomes included pain relief time, adverse events, plasma or serum concentrations of bone metabolic markers like alkaline phosphatase (ALP), blood phosphorus (P), blood calcium ion (Ca^2+^), Estradiol (E2), as well as interleukin 6 (IL-6) in osteoporosis patients. When available data in the articles were insufficient, researchers attempted to contact the corresponding authors by e-mail. No trials were excluded due to their publication status or language, thus reducing the risk of publication bias.

### Selection Criteria

The eligible study should meet the following criteria: (1) RCTs in human; (2) enrolling osteoporosis patients; (3) the RCT studied one of the following comparisons: (i) oral Epimedium in combination with conventional pharmaceutical treatment versus conventional pharmaceutical treatment, (ii) oral Epimedium alone versus conventional pharmaceutical treatment; (4) the clinical endpoints included the outcomes of interest; (5) the RCT reported means and standard deviation (SD) for continuous outcomes, or reported the number of patients analyzed and the number of patients who experienced the event for dichotomous outcomes; (6) the RCT with Jadad score ≥3. Exclusion criteria were as follows: (1) combining Epimedium with other effective ingredients or botanical drugs in Epimedium group as compared to control group; (2) inappropriate outcome measurements; (3) duplicate records; (4) low bone mass (osteopenia) with T-score between −1.0 and −2.5; (5) severe or established osteoporosis with T-score at or below −2.5 with one or more fractures; (6) diabetes-associated osteoporosis or other secondary osteoporosis. The research question of the systematic review and meta-analysis was defined in terms of populations, interventions, comparators, outcomes, and study designs (PICOS) in Table 1.

**TABLE 1:** Populations, interventions, comparators, outcomes, and study designs (PICOS) of the research question.

Parameters	Descriptions
Populations	Osteoporosis patients
Interventions	Epimedium as an alternative or adjuvant medicine
Comparators	Conventional pharmaceutical treatment
Outcomes	Bone mineral density, effective rate, Visual Analog Scale, pain relief time, bone metabolic markers, or adverse events
Study designs	Randomized controlled trials

### Data Extraction and Trial Quality Assessments

Potentially eligible articles were searched and screened based on the inclusion and exclusion criteria by two reviewers (Shihua Shi and Yong Huang) independently. Full texts were retrieved for further evaluation whenever necessary. The following information in eligible studies was rated and extracted: the authors, year of publication, the number of subjects randomized, and the number of subjects analyzed, the age and gender of patients, treatment intervention regimes, duration of treatment, outcome measures, effective rate, and side effects. During the data extraction process, the modified Jadad quality scoring scale was used to assess the quality of the included trials by two researchers (Bonan Chen and Weihao Li) independently. The trial was considered high quality if its score was 4 or higher, and the score of low-quality study was 1–3 points (Miller et al., 1981; Jadad et al., 1996). Finally, consensus was achieved since discrepancies were resolved by joint discussion by the authors.

### Data Synthesis and Statistical Analysis

We carried out most analyses with Review Manager software (Cochrane Collaboration, version 5.4), and Egger’s test was calculated with STATA, version 15 (StataCorp LP, College Station, TX, United States). Meta-analyses were conducted if ≥2 studies could be pooled. Continuous data were expressed as the weighted mean difference (WMD) or standardized mean differences (SMD), and risk ratio (RR) was calculated for dichotomous data. Confidence intervals (CI) was set at 95%. Statistical heterogeneity was assessed using *I* ^*2*^ statistic and Chi^2^ test. Significant heterogeneity was presented when *I* ^2^ > 50% or *p* < 0.1, and random-effect model was utilized to accommodate heterogeneity (Khan et al., 2015). Conversely, fixed-effect model was applied in the absence of substantial heterogeneity (*I* ^2^ ≤ 50% and *p* ≥ 0.1) (Higgins et al., 2003). Two-tailed *p* values less than 0.05 were considered as statistical significance. Subgroup analysis was performed based on study design, dosage form, and duration of the intervention to explore possible causes of heterogeneity among study results. The robustness of merged results was tested with sensitivity analyses by removing individual studies. The risk of publication was assessed by visual analysis of funnel plots when at least 10 studies were eligible in a meta-analysis, and Egger’s test was performed to identify the potential publication bias statistically (Egger et al., 1997). The certainty of evidence was evaluated using the GRADE approach.

## Results

### Selection of Studies

**FIGURE 1:**
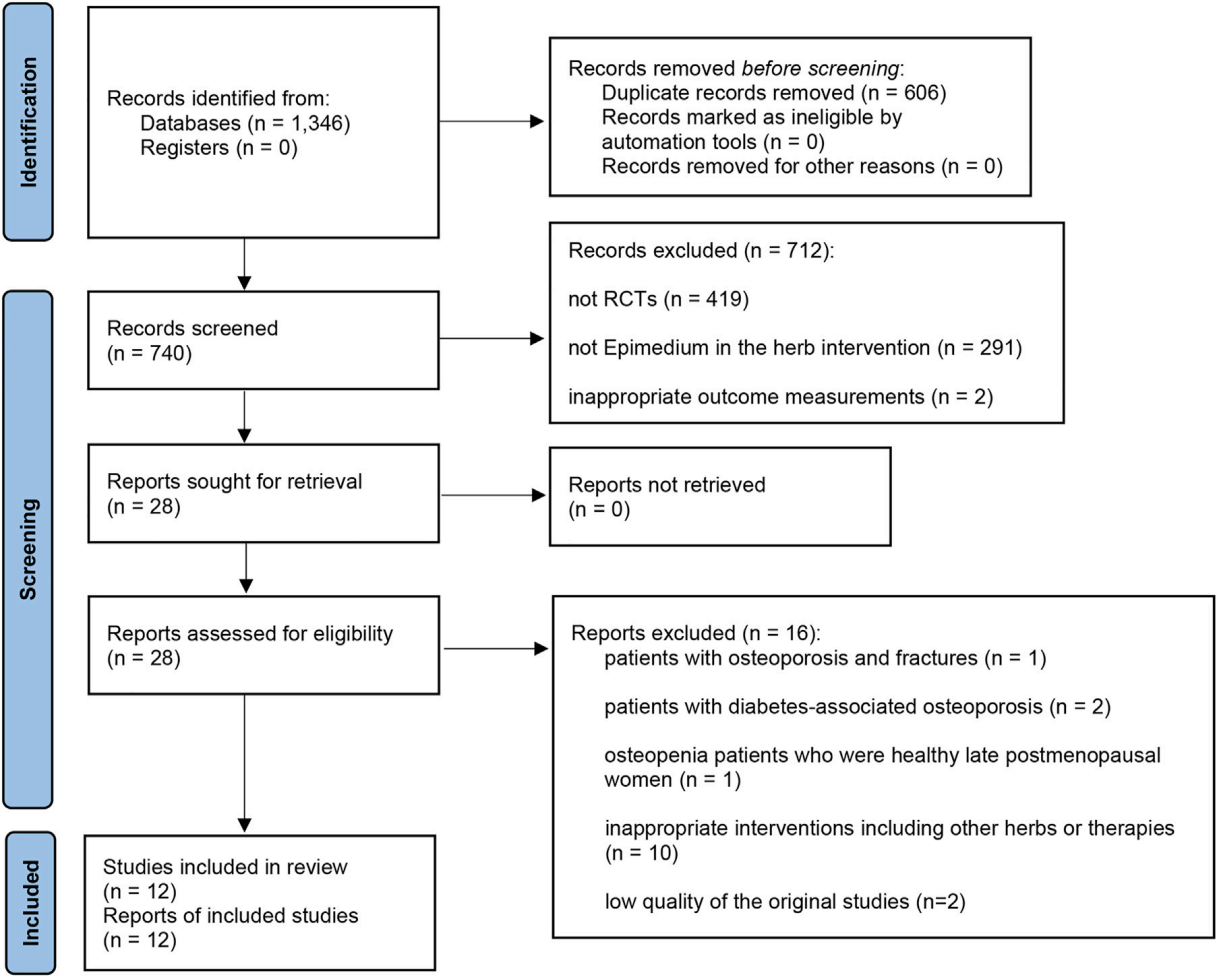
PRISMA2020 flow diagram.

Initially, a sum of 1,346 articles were retrieved as potentially relevant through the database search. Of the 1,346 articles, 606 were duplicates, hence 740 unique publications remained. After the basic researches, reviews, case reports, and the RCTs with intervention rather than Epimedium in the treatment group or reporting irrelevant outcomes were excluded, 28 records were identified after screening via the titles and abstracts based on the inclusion criteria. The full texts of 28 studies were further evaluated, of which 16 articles were removed for the following reasons: patients with osteoporosis and fractures (*n* = 1), patients with diabetes-associated osteoporosis (*n* = 2), osteopenia patients who were healthy late postmenopausal women (*n* = 1), inappropriate interventions including other botanical drugs or therapies (*n* = 10), low modified Jadad scores (*n* = 2). Finally, 12 trials met the inclusion criteria and were included in the qualitative analysis. The screening process was summarized in a PRISMA2020 flow diagram (Figure 1).

### Characteristics and Quality Assessments

All 12 studies included were RCTs published from 2002 to 2019. Seven RCTs comprising of 513 patients compared Epimedium plus conventional pharmacotherapy with conventional pharmacotherapy (Fang, 2013; Wang and Chen, 2013; Zhu et al., 2014a; Zhu et al., 2014b; Dai et al., 2016; Zhou, 2016; Zeng, 2017), and five RCTs comprising of 504 patients compared Epimedium alone with conventional pharmacotherapy (Wang, 2002; Zhao, 2003; Bai, 2009; Zeng et al., 2017; Liu, 2019). Finally, the eligible RCTs represented data on 1,017 subjects. Detailed characteristics of the 12 trials were presented in Table 2. The typical chemical characterization of some effective ingredients of Epimedium was described by Zhang et al. in their study (Zhang et al., 2007). Brevicornu Maxim–derived phytoestrogen flavonoids (EPFs) were fractioned in n-butanol through a series of standardized extraction-isolation procedures under chromatography workstation. The main flavonoid in EPFs was Icariin, which has potential anabolic effects on bones; Genistein and Daidzein within EPFs could activate protein tyrosine phosphatase, induce apoptosis, and inhibit cytokines and membrane depolarization to inhibit osteoclast activity (Zhang et al., 2007). The compositions, concentration, usage, source, and quality control of the preparations in all included studies were shown in Table 3.

**TABLE 2:** Characteristics of the eligible studies included in the meta-analysis.

Study	No. of participants (R/A)	Gender (M/F)	Age (years)	Course of disease (years)	Interventions	Comparison group	Outcomes	Intergroup differences
Wang and Chen (2013)	T:75/75	Data lost	Data lost	NR	plus	Caltrate D (1.5 g calcium carbonate and 125u vitamin D3) 1# qd, p.o	BMD	*p* < 0.05
	C:75/75						VAS	*p* < 0.05
Fang (2013)	T:50/49	T:22/28	T:73.34 ± 6.85	T:12.87 ± 11.36	plus	Alendronate tablet	BMD,	*p* < 0.05
	C:46/44	C:20/26	C:74.12 ± 6.69	C:13.79 ± 10.03		10mg, qd, p.o	ER, Adverse events	*p* < 0.05
Zhu et al. (2014a)	T:40/40	T:12/28	T:58–82 (68.6)	NR	plus	Caltrate D	BMD	*p* < 0.05
	C:40/40	C:13/27	C:56–81 (67.2)			600mg, qd, p.o	PRT	*p* < 0.05
Zhu et al. (2014b)	T:40/40	T:12/28	T:58–82 (68.6)	NR	plus	Caltrate D	ER	*p* < 0.05
	C:40/40	C:13/27	C:56-81(67.2)			600mg, qd, p.o	IL-6	*p* > 0.05
Dai et al. (2016)	T:30/30	0/59	T:56. 97 ± 3. 89	NR	plus	placebo capsules 3# bid, p.o	BMD	NR
	C:30/29		C:56.73 ± 4.26			Caltrate D 600mg, qd, p.o		
Zhou (2016)	T:30/30	0/59	T:56. 97 ± 3. 89	NR	plus	placebo capsules 3# bid, p.o	VAS	NR
	C:30/29		C:56.73 ± 4.26			Caltrate D 600mg, qd, p.o	ER	
Zeng (2017)	T:33/33	T:6/27	65–92	NR	plus	Salmon Calcitonin Injection	BMD	*p* = 0.014
	C:34/34	C:5/29				50iu, i.m	Adverse events	NR
							Ca^2+^	*p* = 0.000
							IL-6	*p* = 0.003
							VAS	*p* = 0.001
Liu (2019)	T:61/61	T:16/45	T:67.46 ± 4.32	T:17.67 ± 2.13	alone	Calcium Carbonate - Vitamin D3 Tablets	BMD	*p* < 0.05
	C:61/61	C:20/41	C:68.66 ± 6.21	C:16.21 ± 1.98		(Ca 600mg/Vitamin D3 125 u)	P	*p* < 0.05
						1# bid, p.o	Ca^2+^	*p* < 0.05
							ALP	*p* < 0.05
Wang (2002)	T:150/150	NR	T:59.5 ± 5.7	NR	alone	Gaitianli (Oyster Shell Calcium Carbonate Chewable Tablets)	BMD, Adverse events	NR
	C:74/74		C:59.5 ± 6.6			200mg, tid, p.o	E2	*p* < 0.01
							Ca^2+^	*p* > 0.05
							ER	*p* < 0.05
Zeng et al. (2017)	T:45/45	T:15/30	T:74.5 ± 3.3	NR	alone	Calcitriol Capsules	BMD	*p* < 0.05
	C:45/45	C:15/30	C:74.3 ± 3.2			0.25ug, bid, p.o	ER	*p* < 0.05
							PRT	*p* < 0.05
							VAS	*p* < 0.05
Bai (2009)	T:28/24	T:0/24	51–63	NR	alone	Alendronate Sodium Tablets	BMD	*p* > 0.05
	C:22/19	C:0/19				70mg, qw, p.o	ALP	*p* > 0.05
							IL-6	*p* > 0.05
							E2	*p* > 0.05
							ER	*p* < 0.01
Zhao (2003)	T:15/15	T:0/15		NR	alone	Premarin (Conjugated equine estrogens)	BMD	NR
	C:10/10	C:0/10	NR			0.625mg, qd, p.o	Ca^2+^	*p* < 0.05
							PRT	NR
							ER	NR
							P	*p* > 0.05
							ALP	*p* < 0.05

R = number of subjects randomized; A = number of subjects analyzed.

ALP, alkaline phosphatase; alone: Epimedium treatment alone; bid, bis in die; Ca^2+^, blood calcium ion; E2, Estradiol; ER, effective rate; g, gramme; IL-6, interleukin 6; im, intramuscular injection; M, months; NR, Not report; P, blood phosphorus; plus: Epimedium treatment plus conventional pharmaceutical treatment (the same as drugs in comparison group); p. o., per os; PRT, pain relief time; qd, quaque die; qw: once a week; T/C, treatment group/control group; tid, ter in die; VAS, visual analog scale.

**TABLE 3:** Compositions, concentration, usage, source, and quality control of Epimedium treatment.

Study	Name of preparation	Composition (Species/Compound) Concentration	Usage	Preparations	Duration Of treatment (M)	Source	Quality control	Chemical analysis
Wang and Chen (2013)	Epimedium decoction	Epimedium 6 g	qd	Decoction	12	Prepared by Wang and Chen (2013)	Hospital Preparation	Based on previous HPLC research
Fang (2013)	Epimedium Extractum Tablet	Epimedium NR	21.6 g tid	Epimedium extract tablet	6	Prepared by Fang (2013)	Hospital Preparation	Based on previous HPLC research
Zhu et al. (2014a)	Epimedium Granules	Epimedium NR	1 g bid	Granules	6	Prepared by Zhu et al. (2014b)	Hospital Preparation	Based on previous HPLC research
Zhu et al. (2014a)	Epimedium Granules	Epimedium NR	1 g bid	Granules	6	Jiangyin Tianjiang Pharmaceutical Co. Ltd.	Prepared according to Chinese pharmacopeia	Based on previous HPLC research
Dai et al. (2016)	Epimedium Capsules	Extract of Epimedium 100 mg	3# bid	Capsules	6	Prepared by Zhou (2016)	Hospital Preparation	Based on previous HPLC research
Zhou (2016)	Epimedium Capsules	Extract of Epimedium 100 mg	3# bid	Capsules	6	Prepared by Zhou (2016)	Hospital Preparation	Based on previous HPLC research
Zeng (2017)	Epimedium treatment	Epimedium 25 g	25 g bid	Granules	1	Prepared by Zeng (2017)	Hospital Preparation	Based on previous HPLC research
Liu (2019)	Extracts of Epimedium	Epimedium 100 g	50 ml bid	Decoction	3	Anhui Bozhou Tengwang Pharmaceutical Co., Ltd.	Prepared according to Chinese pharmacopeia	Based on previous HPLC research
						Lot No. 20140206		Based on previous HPLC research
Wang (2002)	Xianlingpi decoction	*Epimedium koreanum* Nakai 15 g	50 ml bid	Decoction	6	Prepared by Wang (2002)	Hospital Preparation	Based on previous HPLC research
Zeng et al. (2017)	Epimedium treatment	Epimedium 150 g	50 ml tid	Decoction	3	Prepared by Zeng X et al. (2017)	Hospital Preparation	Based on previous HPLC research
Bai (2009)	Epimedium decoction	Epimedium 25 g	50 ml bid	Decoction	6	Prepared by Bai (2009)	Hospital Preparation	Based on previous HPLC research
Zhao (2003)	Epimedium treatment	Epimedium 200 g	1# tid	Decoction	6	Prepared by Zhao (2003)	NR	Based on previous HPLC research

Bid, bis in die; CPT, conventional pharmaceutical treatment (the same as drugs in comparison group); g, gramme; HPLC, high-performance liquid chromatography; M, months; NR, Not report; p.o., per os; qd, quaque die; tid, ter in die.

**FIGURE 2:**
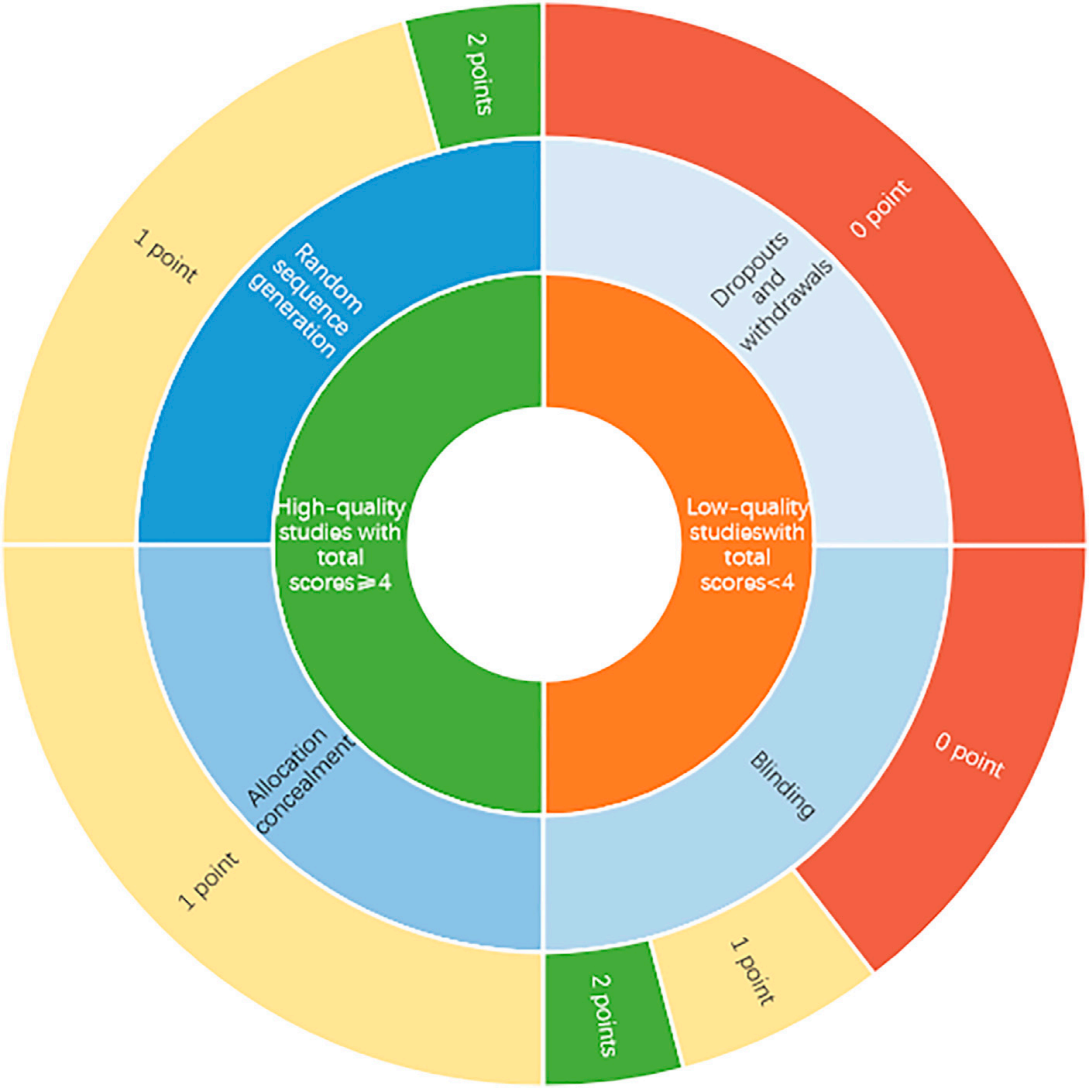
Risk of bias sunburst diagram.

All subjects enrolled were middle-aged and elderly osteoporosis patients. The number of subjects in each RCT varied from 25 to 150. Five studies were of women with postmenopausal osteoporosis (Wang, 2002; Zhao, 2003; Bai, 2009; Dai et al., 2016; Zhou, 2016), seven included a mixed population of men and women (Fang, 2013; Wang and Chen, 2013; Zhu et al., 2014a; Zhu et al., 2014b; Zeng, 2017; Zeng et al., 2017; Liu, 2019). As for the assessment of treatment effect, ten studies used BMD as main measurement (Wang, 2002; Zhao, 2003; Bai, 2009; Fang, 2013; Wang and Chen, 2013; Zhu et al., 2014a; Dai et al., 2016; Zeng, 2017; Zeng et al., 2017; Liu, 2019), and seven studies measured effective rate (Zhao, 2003; Bai, 2009; Fang, 2013; Zhu et al., 2014a; Zhu et al., 2014b; Zhou, 2016; Zeng et al., 2017). Four trials compared Epimedium versus conventional pharmaceutical treatment on VAS (Yang and Zhang, 2010; Wang and Chen, 2013; Zeng, 2017; Zeng et al., 2017). In the case of the methodologic quality of eligible trials, modified Jadad scoring was presented in Table 4 and Figure 2. All included studies mentioned the randomization, of which, Zeng et al. (Zeng et al., 2017) used a random number table. The mean score of the modified Jadad scoring was 3.7. Six trials (Bai, 2009; Fang, 2013; Wang and Chen, 2013; Zhu et al., 2014a; Zhu et al., 2014b; Liu, 2019) scored 3 out of 7 for methodological quality, four (Wang, 2002; Zhao, 2003; Zeng, 2017; Zeng et al., 2017) scored 4 out of 7, and two (Dai et al., 2016; Zhou, 2016) scored 5 out of 7. There were six high-quality trials with an average of 4.3 points (Wang, 2002; Zhao, 2003; Dai et al., 2016; Zhou, 2016; Zeng, 2017; Zeng et al., 2017).

**TABLE 4:** Modified Jadad scale of included trials.

Author (year)	Generation of randomization Allocation sequence (0–2 points)	Randomization allocation Concealment (0–2 points)	Blinding (0–2 points)	Dropouts and withdrawals (0–1 point)	Modified Jadad scale (0–7 point)
Wang and Chen (2013)	1	1	0	1	3
Fang (2013)	1	1	1	1	3
Zhu et al. (2014b)	1	1	0	1	3
Zhu et al. (2014a)	1	1	0	1	3
Dai et al. (2016)	1	1	2	1	5
Zhou (2016)	1	1	2	1	5
Zeng (2017)	2	1	0	1	4
Liu (2019)	1	1	0	1	3
Wang (2002)	1	1	1	1	4
Zeng et al. (2017)	2	1	0	1	4
Bai (2009)	1	1	0	1	3
Zhao (2003)	1	1	1	1	4

### Meta-Analysis

#### Bone Mineral Density

**FIGURE 3:**
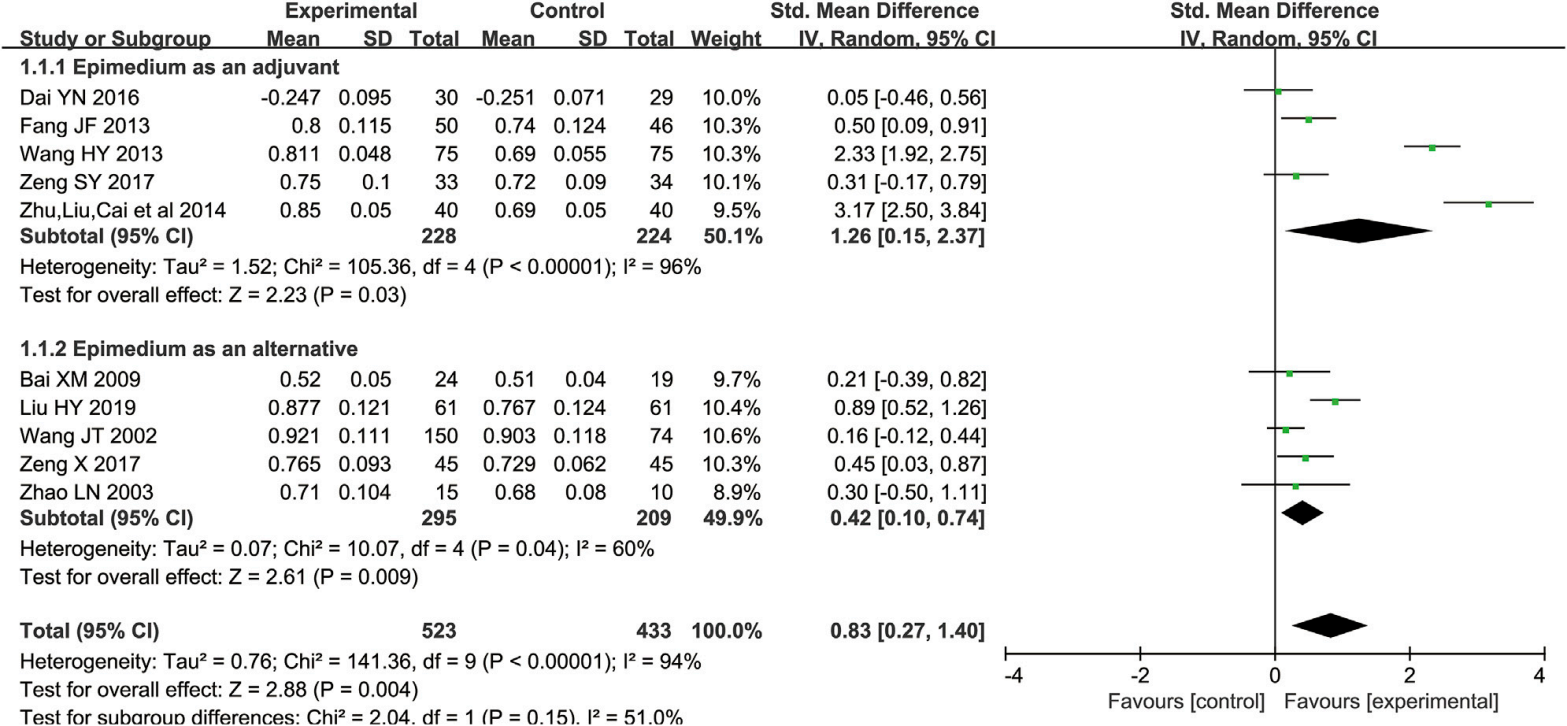
Meta-analysis results of the effect of Epimedium vs conventional pharmacotherapy on bone mineral density.

Ten studies (Wang, 2002; Zhao, 2003; Bai, 2009; Fang, 2013; Wang and Chen, 2013; Zhu et al., 2014a; Dai et al., 2016; Zeng, 2017; Zeng et al., 2017; Liu, 2019) including 956 participants compared Epimedium versus conventional pharmacotherapy according to changes in the BMD. A random effects model was used for statistical analysis since these trials showed heterogeneity in the inconsistency of the trial results, and significant BMD improvement was found in the Epimedium group (SMD = 0.83, 95%CI [0.27, 1.40]; *Z* = 2.88, *p* = 0.004). In the subgroup of Epimedium as an adjuvant (five RCTs including 452 participants), compared with the control arm, this meta-analysis revealed a significant improving effect of Epimedium plus conventional pharmacotherapy on BMD (SMD = 1.26, 95%CI [0.15, 2.37]; *Z* = 2.23, *p* = 0.03). Comparing the effect of Epimedium as an alternative with conventional pharmaceutical treatment through BMD, five trials including 504 participants showed that compared with controls, Epimedium had a better effect on improving BMD (SMD = 0.42, 95%CI [0.10, 0.74]; Z = 2.61, *p* = 0.009), indicating that Epimedium as an alternative therapy may help improve BMD (Figure 3).

#### Clinical Efficacy

**FIGURE 4:**
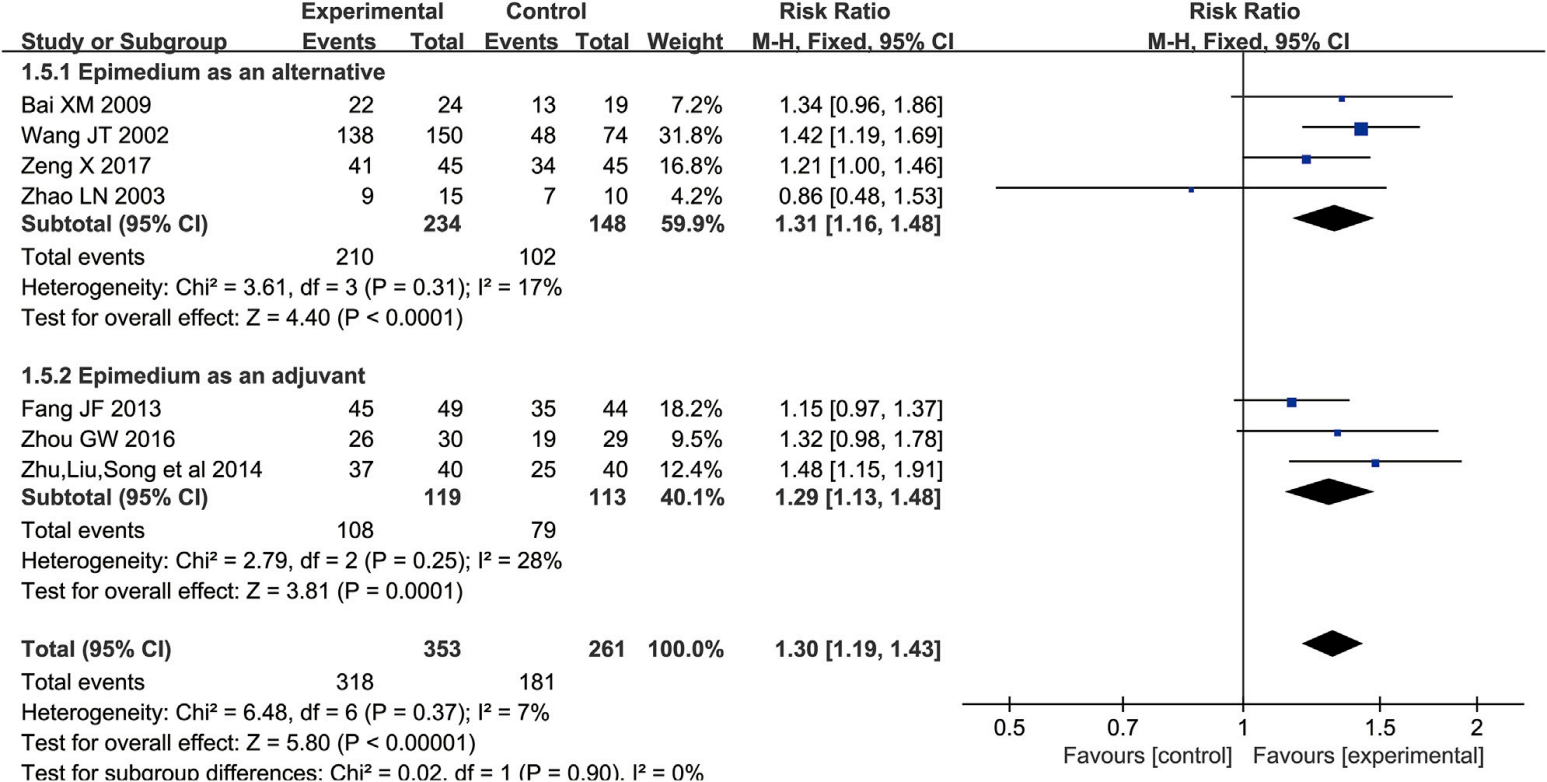
Meta-analysis results of the effect of Epimedium vs conventional pharmacotherapy on clinical efficacy.

Seven trials including 614 participants (Wang, 2002; Zhao, 2003; Bai, 2009; Fang, 2013; Zhu et al., 2014b; Zhou, 2016; Zeng et al., 2017) provided data on effective rate comparing Epimedium plus conventional pharmaceutical treatment with conventional pharmaceutical treatment. Statistical heterogeneity between studies was not observed (*I* ^*2*^ = 7%, *p* = 0.37). Improved effective rate was found in Epimedium group (RR = 1.30 [1.19, 1.43]; *Z* = 5.80, *p* < 0.00001). In the subgroup of Epimedium as an alternative, there were four eligible trials with 382 participants included (Wang, 2002; Zhao, 2003; Bai, 2009; Zeng et al., 2017), which reported Epimedium alone versus conventional pharmaceutical treatment in terms of effective rate. The result showed that effective rate was significantly improved in Epimedium groups (RR = 1.31 [1.16, 1.48]; *Z* = 4.40, *p* < 0.0001). In the subgroup of Epimedium as an adjuvant (Fang, 2013; Zhu et al., 2014b; Zhou, 2016), compared to conventional pharmacotherapy, Epimedium plus conventional pharmacotherapy may significantly improve the effective rate (n = 232; RR = 1.29 [1.13, 1.48]; *Z* = 3.81, *p* = 0.0001), suggesting that Epimedium, as an adjunctive treatment, contributed to improving clinical effects of treatment in patients with osteoporosis (Figure 4).

#### Visual Analog Scale

**FIGURE 5:**
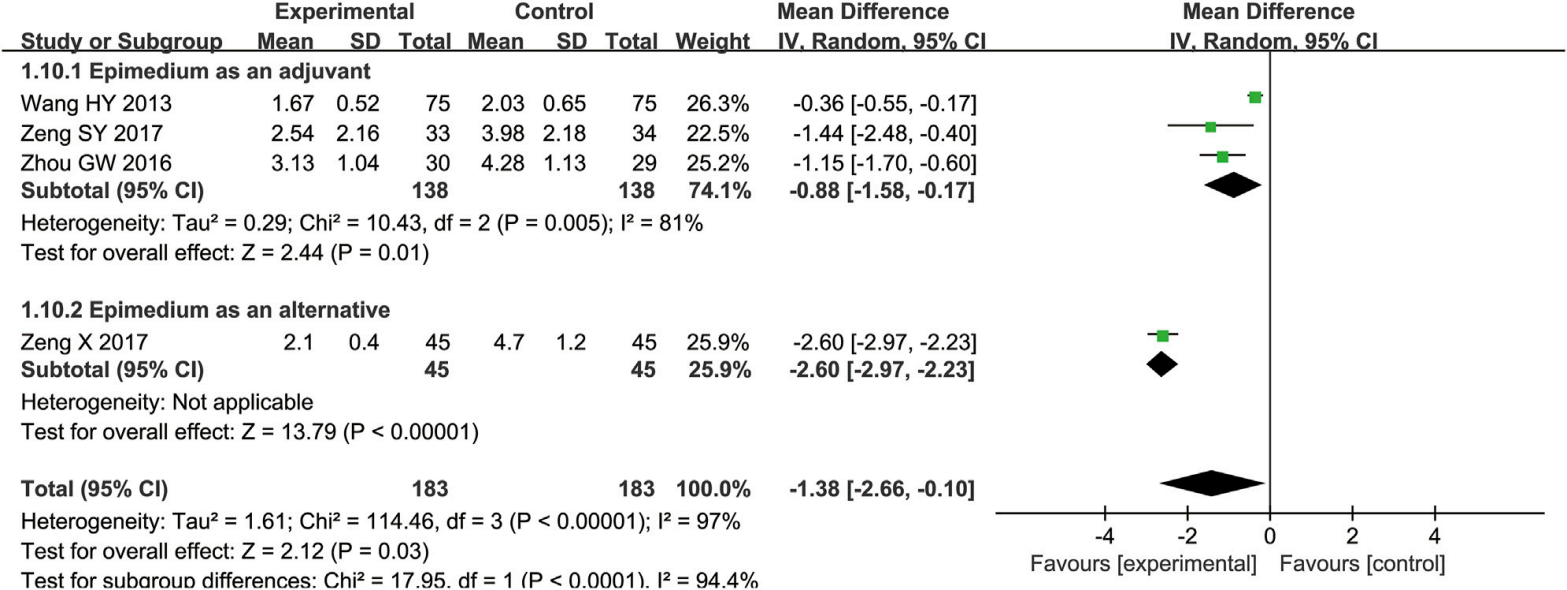
Meta-analysis results of the effect of Epimedium vs conventional pharmacotherapy on VAS.

There were four RCTs with 366 participants included (Wang and Chen, 2013; Zhou, 2016; Zeng, 2017; Zeng et al., 2017), comparing Epimedium versus conventional pharmacotherapy according to changes in the VAS pain scores. Epimedium was superior in decreasing VAS (WMD = −1.38, 95% CI [−2.66, −0.10], *Z* = 2.12, *p* = 0.03). Similar results were found in the subgroup of Epimedium as an alternative (WMD = −2.60, 95% CI [−2.97, −2.23], *Z* = 13.79, *p* < 0.00001). In terms of Epimedium as an adjuvant, similar results were also found (n = 276; WMD = −0.88, 95% CI [−1.58, −0.17], *Z* = 2.44, *p* = 0.01) (Figure 5).

#### Pain Relief Time

Only one RCT (Zhu et al., 2014a) investigated and reported the effects of Epimedium as an adjuvant versus conventional pharmaceutical treatment on the pain relief time. The Epimedium group used Epimedium granules plus conventional pharmaceutical treatment for 6 months, and the study showed that compared with conventional pharmacotherapy, Epimedium granule plus conventional pharmacotherapy was superior in shortening pain relief time. In terms of Epimedium as an alternative, only one RCT (Zhao, 2003) available reported Epimedium alone on pain relief time without the data on standard deviation, and the relevant meta-analysis was not conducted because of the limited number of eligible RCTs and insufficient data.

#### Biochemical Markers

##### Alkaline Phosphatase

**FIGURE 6:**
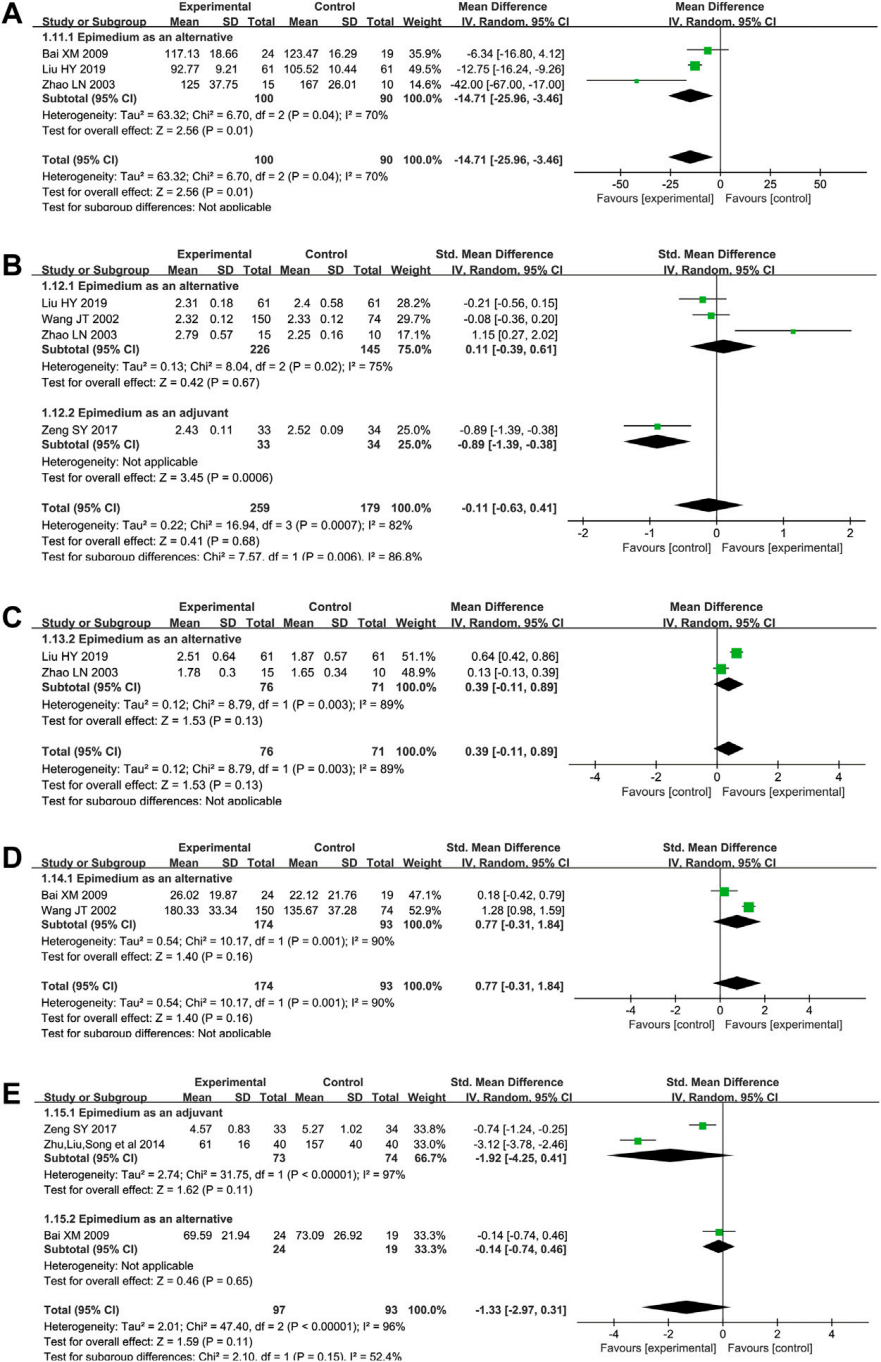
Meta-analysis results of biochemical markers. **(A)** Alkaline phosphatase **(B)** Calcium ion **(C)** Phosphorus **(D)** Estradiol **(E)** IL-6.

Three RCTs reported the effect of Epimedium decoction as an alternative on ALP (Zhao, 2003; Bai, 2009; Liu, 2019). The pooled data showed that compared with conventional pharmacotherapy, Epimedium decoction used alone was superior in decreasing ALP than conventional pharmaceutical treatment (WMD = −14.71, 95% CI [−25.96, −3.46], Z = 2.56, *p* = 0.01; random effect model) (Figure 6A). In terms of Epimedium as an adjuvant, there was not RCT available reported Epimedium on ALP, and the relevant meta-analysis could not be conducted.

##### Ca^2+^

Four RCTs (Wang, 2002; Zhao, 2003; Zeng, 2017; Liu, 2019) provided data on Ca^2+^ comparing Epimedium with conventional pharmaceutical treatment. The pooled analysis did not show any significant difference in Ca^2+^ between the Epimedium group and the control group (n = 438; SMD = −0.11 [−0.63, 0.41]; *Z* = 0.41, *p* = 0.68; random effect model) (Figure 6B). In the subgroup of Epimedium as an alternative, same results were found in three included studies (Wang, 2002; Zhao, 2003; Liu, 2019) (n = 371; SMD = 0.11 [−0.39, 0.61]; *Z* = 0.42, *p* = 0.67; random effect model).

##### P

The effect of oral Epimedium on P was controversial, and there was a paucity of RCTs comparing Epimedium with conventional pharmaceutical treatment on this outcome. On the one hand, one study reported Epimedium as an alternative significantly improved the level of P than control group (2.51 ± 0.64 versus 1.87 ± 0.57) (Liu, 2019). On the other hand, Zhao showed Epimedium treatment observed no difference in P, which was 1.78 ± 0.30 versus 1.65 ± 0.34 (Zhao, 2003). The pooled analysis did not show any significant difference in P between the Epimedium group and the control group (WMD = 0.39 [-0.11, 0.89]; *Z* = 1.53, *p* = 0.13; random effect model) (Figure 6C).

##### E2

Oral Epimedium alone was significantly more effective than conventional pharmaceutical treatment in the two RCTs investigating and reporting the effects of Epimedium on E2 (Wang, 2002; Bai, 2009), but the meta-analysis could not detect any significant between-group difference (SMD = 0.77, [−0.31, 1.84]; *Z* = 1.40, *p* = 0.16; random effect model). Subgroup analysis was not conducted due to the inadequate number of the eligible studies. (Figure 6D).

##### IL-6

Three RCTs (Bai, 2009; Zhu et al., 2014b; Zeng, 2017) investigated and reported the effects of Epimedium versus conventional pharmaceutical treatment on IL-6. The pooled analysis found no convincing results, and did not show any significant difference in IL-6 between the Epimedium and the conventional pharmacotherapy groups (SMD = −1.33 [−2.97, 0.31]; *Z* = 1.59, *p* = 0.11; random effect model). In terms of Epimedium as an adjuvant, similar results were found after pooling two available trials (Zhu et al., 2014b; Zeng, 2017) (SMD = -1.92 [-4.25, 0.41]; *Z* = 1.62, *p* = 0.11; random effect model) (Figure 6E).

#### Adverse Events

Three studies (Wang, 2002; Fang, 2013; Zeng, 2017) reported that no adverse events occurred during the treatments. Other studies comparing Epimedium versus conventional pharmacotherapy did not report adverse events. It was a pity that the safe issue of Epimedium treating osteoporosis cannot be evaluated by meta-analysis, and it was not feasible to determine the safety of Epimedium plus conventional pharmaceutical treatment based on the available RCTs, though Epimedium was non-toxic according to cellular and animal studies.

#### Sensitivity Analyses

The overall results of leave-one-out sensitivity analyses on BMD, effective rate, Ca^2+^, and IL-6 in RCTs comparing Epimedium with conventional pharmacotherapy remained similar with the pooled results of included studies, and were not reversed by deleting any study, which supported that the results of meta-analysis above were stable with good consistency. However, the results of VAS, ALP, E2, and blood phosphorus were not stable in sensitivity analyses, indicating a low level of comparability between studies. Data for sensitivity analysis were showed in Table 5. The reasons for the heterogeneity may come from clinical heterogeneity that was common in RCTs studying botanical drugs including dosage forms and the duration of the intervention, and thus, the medicinal strength was not consistent among individual studies.

**TABLE 5:** Summarized data for sensitivity analysis.

Outcome	Study	Data with study removed	I2 (%)	Test for overall effect	
		RR/MD/SMD (95%CI)		Z	*p*
BMD	Dai et al. (2016)	0.92 (0.31, 1.53)	94	2.96	0.003
	Fang (2013)	0.87 (0.23, 1.51)	94	2.67	0.008
	Wang and Chen (2013)	0.65 (0.18, 1.12)	89	2.70	0.007
	Zeng (2017)	0.89 (0.27, 1.51)	94	2.81	0.005
	Zhu et al. (2014a)	0.59 (0.11, 1.07)	91	2.40	0.02
	Bai (2009)	0.90 (0.29, 1.51)	94	2.88	0.004
	Liu (2019)	0.82 (0.18, 1.47)	94	2.50	0.01
	Wang (2002)	0.91 (0.28, 1.54)	93	2.82	0.005
	Zeng et al. (2017)	0.87 (0.24, 1.51)	94	2.70	0.007
	Zhao (2003)	0.88 (0.28, 1.49)	94	2.87	0.004
Effective rate	Bai (2009)	1.30 (1.19, 1.43)	22	5.54	<0.00001
	Wang (2002)	1.25 (1.13, 1.39)	0	4.27	<0.0001
	Zeng (2017)	1.32 (1.20, 1.46)	16	5.47	<0.00001
	Zhao (2003)	1.32 (1.21, 1.45)	0	6.06	<0.00001
	Fang (2013)	1.34 (1.21, 1.48)	0	5.57	<0.00001
	Zhou (2016)	1.30 (1.19, 1.43)	22	5.51	<0.00001
	Zhu et al. (2014b)	1.28 (1.16, 1.41)	0	5.04	<0.00001
Visual Analog Scale	Wang and Chen (2013)	−1.77 (−2.85, −0.68)	90	3.19	0.001
	Zeng (2017)	−1.37 (−2.88, 0.15)	98	1.77	0.08
	Zhou (2016)	−1.46 (−3.18, 0.26)	98	1.67	0.10
	Zeng et al. (2017)	−0.88 (−1.58, −0.17)	81	2.44	0.01
ALP	Bai (2009)	−24.65 (−52.81, 3.51)	81	1.72	0.09
	Liu (2019)	−22.29 (−57.04, 12.46)	85	1.26	0.21
	Zhao (2003)	−11.52 (−16.47, −6.57)	23	4.56	<0.00001
Blood calcium ion (Ca2+)	Liu (2019)	−0.02 (−0.87, 0.83)	88	0.04	0.97
	Wang (2002)	−0.06 (−0.94, 0.82)	88	0.14	0.89
	Zhao (2003)	−0.35 (−0.76, 0.07)	74	1.64	0.1
	Zeng et al. (2017)	0.11 (−0.39, 0.61)	75	0.42	0.67
Blood phosphorus	Liu (2019)	0.13 (−0.13, 0.39)	Not applicable	0.98	0.33
(P)	Zhao (2003)	0.64 (0.42, 0.86)	Not applicable	5.83	<0.00001
Estradiol (E2)	Bai (2009)	1.28 (0.98, 1.59)	Not applicable	8.3	<0.00001
	Wang (2002)	0.18 (−0.42, 0.79)	Not applicable	0.6	0.55
IL-6	Zeng et al. (2017)	−1.63 (−4.55, 1.29)	98	1.09	0.27
	Zhu et al. (2014a)	−0.47 (−1.05, 0.12)	56	1.56	0.12
	Bai (2009)	−1.92 (−4.25, 0.41)	97	1.62	0.11

#### Subgroup Analysis

**FIGURE 7:**
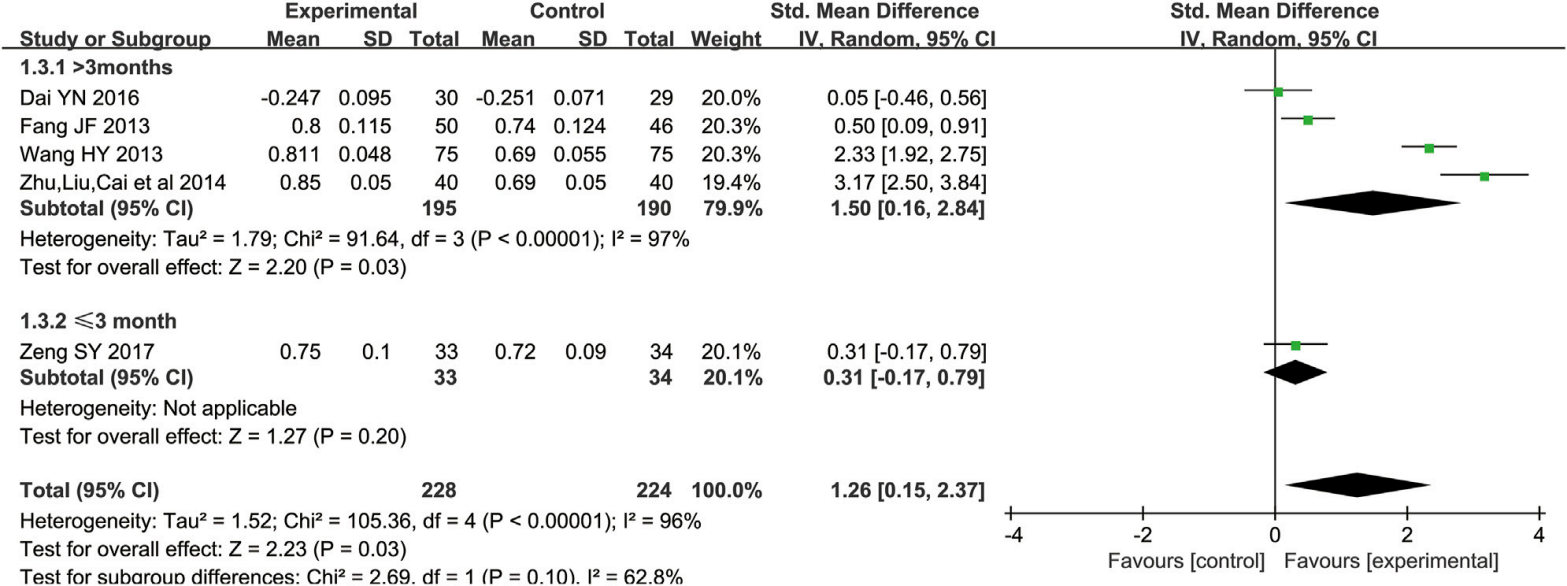
Subgroup analyses for Epimedium as an adjuvant on the duration of the intervention.

**FIGURE 8:**
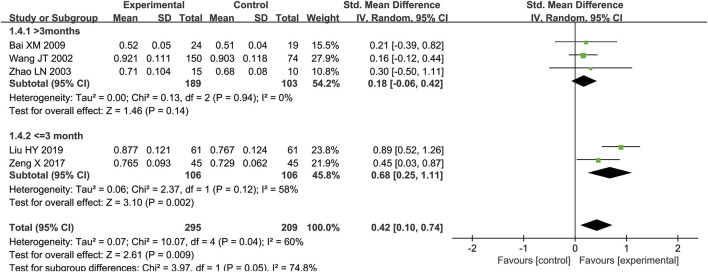
Subgroup analyses for Epimedium as an alternative on the duration of the intervention.

Subgroup analyses were conducted for dosage forms (Epimedium granules, Epimedium decoction, or extracts of Epimedium) and duration of the intervention (≤3 months or >3 months) when two or more RCTs were included (Table 6). Results of subgroup analyses yielded that when the duration of Epimedium plus conventional pharmacotherapy was more than 3 months, more benefits on BMD were identified (*p* = 0.03). More benefits on BMD were identified when the duration of Epimedium as an alternative was ≤3 months (*p* = 0.002). The subgroup analysis about the duration of the intervention may give some hints on the best treatment course, suggesting that the recommended duration of Epimedium as an adjuvant was >3 months (Figure 7), and the recommended duration of Epimedium as an alternative was ≤3 months (Figure 8). In subgroup analyses, we also found that BMD might be associated with the dosage form of Epimedium, as Epimedium decoction plus conventional pharmacotherapy brought more benefits (SMD = 2.33 [1.92, 2.75]) compared with Epimedium granules (SMD = 1.73 [-1.07, 4.53]), extractum tablet of Epimedium (SMD = 0.50 [0.09, 0.91]), or Epimedium capsules (SMD = 0.05 [−0.46, 0.56]).

**TABLE 6:** Summarized results of subgroup analyses.

Outcomes	Experimental group	Control group	Subgroup	Trial (n)	Sample size (n)	Effect estimate WMD/RR (95%CI)	*I2*	*p*
BMD	plus	CPT or CPT plus Epimedium placebo	Dosage forms	—	—	—	—	—
			Epimedium granules	2	147	1.73 (−1.07, 4.53)	98%	<0.00001
			Epimedium decoction	1	150	2.33 (1.92, 2.75)	NA	<0.00001
			Extractum Tablet of Epimedium	1	96	0.50 (0.09, 0.91)	NA	0,02
			Epimedium capsules	1	59	0.05 (−0.46, 0.56)	NA	0.86
			Duration of the intervention	—	—	—	—	—
			≤3 months	1	67	0.31 (−0.17, 0.79)	NA	0.2
			>3 months	4	385	1.50 (0.16, 2.84)	97%	0.03
	alone	CPT	Dosage forms	—	—	—	—	—
			Epimedium granules	0	0	NA	NA	NA
			Epimedium decoction	5	504	0.42 (0.10, 0.74)	60%	0.009
			Extractum Tablet of Epimedium	0	0	NA	NA	NA
			Epimedium capsules	0	0	NA	NA	NA
			Duration of the intervention	0	—	—	—	—
			≤3 months	2	212	0.68 (0.25, 1.11)	58%	0.002
			>3 months	3	292	0.18 (−0.06, 0.42)	0%	0.14
Effective rate	plus	CPT or CPT plus Epimedium placebo	Dosage forms	—	—	—	—	—
			Epimedium granules	1	80	1.48 (1.15, 1.91)	NA	0.003
			Epimedium decoction	0	0	NA	NA	NA
			Extractum Tablet of Epimedium	1	93	1.15 (0.97, 1.37)	NA	0.1
			Epimedium capsules	1	59	1.32 (0.98, 1.78)	NA	0.07
			Duration of the intervention	—	—	—	—	—
			≤3 months	0	0	NA	NA	NA
			>3 months	3	232	1.29 (1.13, 1.48)	28%	0.0001
	alone	CPT	Dosage forms	—	—	—	—	—
			Epimedium granules	0	0	NA	NA	NA
			Epimedium decoction	4	382	1.31 (1.16, 1.48)	17%	<0.0001
			Extractum Tablet of Epimedium	0	0	NA	NA	NA
			Epimedium capsules	0	0	NA	NA	NA
			Duration of the intervention	—	—	—	—	—
			≤3 months	1	90	1.21 (1.00, 1.46)	NA	NA
			>3 months	3	292	1.35 (1.16, 1.57)	25%	<0.0001

Alone: epimedium treatment alone; NA, not applicable; plus: epimedium treatment plus conventional pharmaceutical treatment (the same as drugs in comparison group).

#### Risk of Publication Bias

**FIGURE 9:**
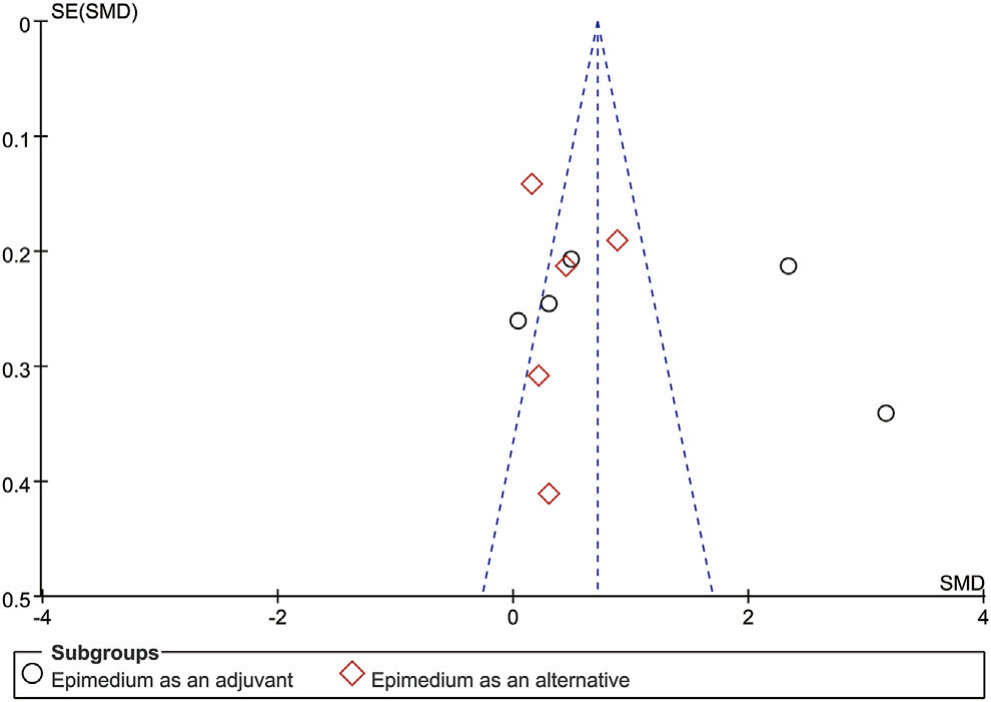
Funnel plot of outcome of BMD.

**FIGURE 10:**
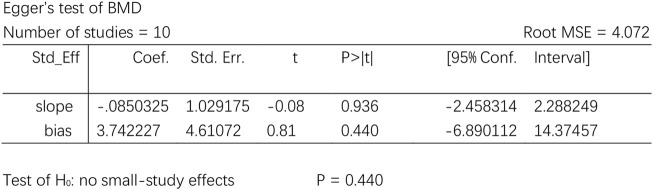
The publication bias analysis.

As BMD was reported by 10 RCTs, the publication bias was analyzed by funnel plots, with the SMD value as the horizontal coordinate and SE (SMD) as the longitudinal coordinates. Although light publication bias was noted visually by the funnel plot of BMD (Figure 9), a respectable *p* value was detected when the Egger’s test was conducted to test the significance of funnel plot asymmetry. No strong evidence was detected for a publication bias based on statistical tests (t = 0.81, *p* = 0.440) (Figure 10).

#### The Certainty of Evidence

The certainty of findings from RCTs relevant to the effective rate of Epimedium for osteoporosis patients was high. The quality of evidence relevant to RCTs reporting the effect of Epimedium on BMD, VAS, Ca^2+^, P, and E2 was moderate. However, the certainty of evidence about the effect of Epimedium on ALP and IL-6 was low. The GRADE evidence profile for all outcomes was illustrated in Supplementary Table S1.

## Discussion

### Summary of Results

Osteoporosis has developed into a globally challenging disease that causes large-scale clinical and socioeconomic problems, affecting mounting patients worldwide (Arcos et al., 2014). Interventions to abate the progression of osteoporosis and to prevent fractures were necessary. Epimedium has been safely used for thousands of years and is widely used for osteoporosis in Asia nowadays. Botanical drugs, including Epimedium, are acknowledged in osteoporosis treatment (Ma et al., 2019). According to the Pharmacopoeia of the People’s Republic of China (Commission, 2015), Epimedium used in osteoporosis was aimed at the basic pathogenesis of osteoporosis, yang-deficiency of the kidney, which causes aching and flaccidity of the bones. Epimedium with the function of reinforcing kidney was helpful for promoting the growth of skeleton, based on the theory of TCM. The modern usage of Epimedium in osteoporosis is a paradigm integrated traditional Chinese and western medicine. In the present study based on “WE” medicine, we evaluated the efficacy of oral Epimedium as single botanical drug in treating osteoporosis, and we demonstrated that oral Epimedium as an adjunctive or alternative treatments might have positive impacts on VAS, effective rate, and BMD for osteoporosis patients. The role Epimedium played during osteoporosis may be therapeutic effect rather than placebo effect, since the increase in BMD suggested that Epimedium might provide a method of restoring skeletal integrity in osteoporosis patients. Besides, oral Epimedium as an alternative may have a positive impact on decreasing ALP level. We have conducted comprehensive meta-analysis of oral Epimedium as single botanical drug on BMD, VAS, and bone metabolic markers during osteoporosis based on recent developments and “WE” medicine, investigating the potential mode of action of Epimedium, and confirming the therapeutic effect of Epimedium.

While it is well-known that single botanical drug is a medicine made of many ingredients, the effect of Epimedium as single botanical drug has been underestimated and the present study has met with a lot of suspicions. Fortunately, the results based on “WE” medicine will speak for themselves. In TCM, Epimedium used traditionally as a botanical drug in osteoporosis conforms with the basis pathogenesis of osteoporosis, which is kidney asthenia and dates back to the Han dynasty (Wan et al., 2019). Recent preclinical studies, both *in vitro* (Zhai et al., 2014) and *in vivo* (Li et al., 2014), have demonstrated that Epimedium contains various compounds pharmacologically effective in preventing osteoporosis. The anti-osteoporotic mechanisms of Epimedium were most likely associated with several neuropeptides involved in regulation of the brain/spinal cord/bone axis (Liu et al., 2018), which provided theory basis for the clinic use of Epimedium. However, some physicians still believed that it was impossible for Epimedium to be used as a single botanical drug to treat osteoporosis. Some researchers used to believe that the effect of single botanical drug, especially Epimedium, was placebo effect, and the main effective outcomes may be caused by conventional pharmaceutical treatment, especially when the botanical drug was used as an adjuvant. Intriguingly, clinical results supporting the use of Epimedium as a single botanical drug for osteoporosis have been accumulating recently (Zhang et al., 2007), contributing to the conduction of the present study suggesting that Epimedium might be effective when it was used even as an alternative.

The ability to generalize and translate results to clinical practice is a challenge for botanical drugs studies, and the pooled evidence for Epimedium is needed for better clinical practice. BMD is the primary therapeutic target and direct efficacy indicator for osteoporosis treatment (Villareal et al., 2001). However, BMD could not be changed significantly in a short time based on bone biology. Since BMD as the sole measurement of treatment effect may not be sensitive enough, the clinical efficacy was also utilized as outcome. Given that the relief of clinical symptoms, like severe ostealgia, can greatly improve quality of life and were adopted as a main outcome measurement in many studies, we also analyzed pain VAS scores, which could be used to reflect the effect of Epimedium treatment on osteoporosis in a shorter duration (Giannini et al., 2021). Based on our results, Epimedium as an adjunctive treatment might decrease VAS. Therefore, when the role of conventional pharmacotherapy was not satisfying, especially the analgesic effect, osteoporosis patients might choose Epimedium in conjunction with the basic drug treatment to achieve pain relief.

As regards Epimedium used alone, Epimedium may improve the biochemical markers of bone metabolism and inhibit the ALP activity as an alternative. The serum ALP was significantly attenuated in Epimedium group compared with conventional pharmacotherapy, demonstrating a decrease in bone resorption (Indran et al., 2016). In addition, Epimedium used alone may be beneficial for improving BMD, and decreasing VAS. Hence, if the patients cannot tolerate the side effects of conventional pharmaceutical treatments, they might use Epimedium treatment to improve BMD, relieve pain, and improve the biochemical markers of bone metabolism. Nevertheless, the pain relief time was excluded from quantitative analysis of Epimedium due to few studies reporting on it. The reason why few studies reported this outcome might be that the time that Epimedium played effect might not be as fast as conventional pharmaceutical treatment, which could be also speculated by the course of Epimedium, which was no less than 1 month in all included trials. Epimedium may not have an advantage on shortening the pain relief time, though Epimedium was superior on decreasing VAS. What’s more, the course of Epimedium treatment as a single botanical drug was not supposed to be too short during osteoporosis, and maintenance of Epimedium treatment for at least 1 month might be recommended based on the present studies. Nonetheless, the result is still under debate because the optimal duration of Epimedium treatment has not yet been thoroughly investigated. Additionally, reports on safety issues of Epimedium were limited. Considering the treatment duration of osteoporosis, more attention should be paid to safety profiles. Detailed and long-term safety studies should be conducted.

As far as phytoestrogens’ effect on bone metabolism is concerned, previous studies have illustrated that Epimedium exhibited a certain estrogen-like effect, and Icariin, the main effective component of Epimedium brevicornum Maxim, which is used as the chemical marker for standardization of the quality of Epimedium extracts based on Chinese Pharmacopeia, may promote the secretion of E2 by granulosa cells (Li and Shi, 2021). According to the individual RCT, Epimedium as an adjuvant might improve E2 significantly in clinical practice, while the meta-analysis could not detect convincing results with significant between-group difference, which may be induced by the paucity of data on the E2 in the osteoporosis population treated with Epimedium. Additionally, the serum calcium levels indicated no change in the Epimedium group compared with conventional pharmacotherapy, which may suggest that Epimedium, a rich source of phytoestrogen lignan, did not affect serum calcium. Similar to our findings, Zhang et al. reported that a group of flavonoids derived from Epimedium inhibited bone resorption and stimulated bone formation through a pathway independent of intestinal calcium absorption (Zhang et al., 2006). Unfortunately, Epimedium did not achieve the desired effect on blood phosphorus and IL-6. The efficacy of Epimedium was not superior to conventional pharmaceutical treatment on these bone metabolism outcomes, which were usually omitted in RCTs, with a limited number of relevant trials.

### Strengths and Limitations

Firstly, although Epimedium contained in many herbal formulas has been widely used to treat osteoporosis, Epimedium as single botanical drug was not involved in clinical practice guidelines for the pharmacological management of osteoporosis (Eastell et al., 2019), and patients who benefit from Epimedium treatment have not achieved globalization. The results of the present study would benefit more osteoporosis patients all over the world, making osteoporosis treatments work better, considering that osteoporosis remains a health concern worldwide. Secondly, the discoveries from the systematic review and meta-analysis are meaningful considering that providing evidence-based guideline recommendations according to results of RCTs is of vital importance to clinical decision-making. Based on our results, osteoporosis patients might choose conventional pharmacotherapy plus Epimedium to achieve better pain relief with BMD improvement. Conventional pharmacotherapy plus Epimedium may have the potential to develop into a new standard combination therapy complementing the international osteoporosis guideline available. Impressively, osteoporosis patients might have another choice which might be Epimedium treatment to improve BMD, relieve pain, and regulate bone metabolism if they cannot tolerate the side effects of conventional pharmacotherapy. Thirdly, the systematic review included a balanced, comprehensive, and critical view of the literature in the field. After the comprehensive search strategies were conducted, we reported not only positive results but negative results objectively in the present study. Apart from BMD, we also systematically reviewed VAS, pain relief time, adverse events, and bone metabolic markers like ALP, P, Ca^2+^, and E2 as well as IL-6 to achieve a comprehensive understanding of the effect of Epimedium against osteoporosis and identify the therapeutic potential of Epimedium for treating osteoporosis. Fourthly, the present study was clearly defined to identify, categorize, analyze, and report the aggregated clinical trials of osteoporosis treated with oral Epimedium, and embodied “WE” medicine, which could meet some of the current unmet medical needs (Cheng and Belli, 2020).

Admittedly, our study has some limitations. First, a few included studies were of low methodological quality. Appropriate quality control is warranted in future studies, since the meta-analytical approach does not directly collect data in the frontline of the clinical research setting, and its reliability depends on the individual clinical studies. Second, the frequency of osteoporosis varies in different races and regions. For instance, white women have higher osteoporotic fracture risks than black women, and northern Europe and Mediterranean areas experience the highest rates and the lowest rates respectively (Johnel et al., 1992). Based on these differences, the benefits of Epimedium may need to compare different ethnic groups and different regions in the future. Third, some original studies did not report the complete species and drug name of Epimedium, and we had to use Epimedium on the composition (Species/Compound) part in Table 3. This is a particular issue in Epimedium where many species are potentially used, and the full species name including authorities and family needs to be included in the original studies (Rivera et al., 2014). Fourth, a common problem of studying Epimedium treatment is the difficulty of quantifying the dosage. The dosages of Epimedium used in the included trials were not all the same. The best dosage of Epimedium treating osteoporosis and reference dosage need more explorations. Fifth, a vital challenge for Epimedium was the differences in the formulation and chemical constituents and thus, the medicinal strength was not consistent among individual studies. To produce a consistent product for clinical studies and reduce the heterogeneity, researchers are supposed to address the differences in extraction and processing technologies, and find the optimal formulation. Sixth, adequate clinical data supporting the mechanisms of Epimedium on osteoporosis including neural/neuropeptides/hormonal/bone axis were vastly missing. Only E2 levels were analyzed in the present study since other relevant data were not available in RCTs. Detailed studies incorporating a careful design and meticulous execution about the potential benefits and mechanisms of Epimedium on osteoporosis are clearly warranted to enable further evidence-based development of natural products using a rigorous scientific approach (Heinrich et al., 2020).

## Conclusions

In conclusion, this systematic review and meta-analysis revealed that Epimedium administered orally as an adjuvant or alternative might have positive impacts on BMD, effective rate, and VAS for osteoporosis patients. Furthermore, Epimedium might regulate bone metabolism when it is used as an alternative, with the recommended treatment duration ≤3 months. The recommended treatment duration is more than 3 months when Epimedium is used as an adjuvant. Given its demonstrated effects, Epimedium administration, especially Epimedium decoction, as a new avenue in the treatment of osteoporosis is worth exploring furtherly. However, considering the intrinsic limitations of the included trials, higher quality RCTs with longer follow-ups are warranted to confirm the results above.

## Data Availability

The original contributions presented in the study are included in the article/Supplementary Material, further inquiries can be directed to the corresponding author.
