# Supplementary material for: Epimedium for Osteoporosis Based on Western and Eastern Medicine: An Updated Systematic Review and Meta-Analysis
Source: Front Pharmacol. 2022 Mar 31;13:782096. doi: 10.3389/fphar.2022.782096 (PMC9008843; doi:10.3389/fphar.2022.782096)
Supplement: Supplementary file 2 [file Table2.DOCX]

**Search strategies**

| Literature databases | Search items | Items found |
| --- | --- | --- |
| (1) PubMed | Search: ((("Epimedium"[MeSH Terms]) OR ((((((((((("Epimedium"[Title/Abstract]) OR ("Epimediums"[Title/Abstract])) OR ("Epimedium sagittatum"[Title/Abstract])) OR ("Epimedium sagittatums"[Title/Abstract])) OR ("sagittatum, Epimedium"[Title/Abstract])) OR ("sagittatums, Epimedium"[Title/Abstract])) OR ("Epimedium grandiflorum"[Title/Abstract])) OR ("Epimedium grandiflorums"[Title/Abstract])) OR ("grandiflorum, Epimedium"[Title/Abstract])) OR ("grandiflorums, Epimedium"[Title/Abstract])) OR ("Epimedii Folium"[Title/Abstract]))) AND (((((((((((((((("Osteoporosis"[Title/Abstract]) OR ("Osteoporosis, Senile"[Title/Abstract])) OR ("Osteoporoses, Senile"[Title/Abstract])) OR ("Senile Osteoporoses"[Title/Abstract])) OR ("Senile Osteoporosis"[Title/Abstract])) OR ("Osteoporosis, Age Related"[Title/Abstract])) OR ("Bone Loss, Age-Related"[Title/Abstract])) OR ("Age-Related Bone Loss"[Title/Abstract])) OR ("Age-Related Bone Losses"[Title/Abstract])) OR ("Bone Loss, Age Related"[Title/Abstract])) OR ("Bone Losses, Age-Related"[Title/Abstract])) OR ("Age-Related Osteoporosis"[Title/Abstract])) OR ("Age Related Osteoporosis"[Title/Abstract])) OR ("Age-Related Osteoporoses"[Title/Abstract])) OR ("Osteoporoses, Age-Related"[Title/Abstract])) OR ("Osteoporosis"[MeSH Terms]))) AND ("randomized") | 7 |
| (2) Science Direct | #1  Title, abstract, keywords: “Epimedium” OR “Epimedium sagittatum” OR “sagittatum, Epimedium” OR “sagittatums, Epimedium” OR “Epimedium grandiflorum” OR “Epimedium grandiflorums” OR “grandiflorum, Epimedium” OR “grandiflorums, Epimedium” OR “Epimedii Folium”  #2  Title: “Osteoporosis” OR “Osteoporoses, Senile” OR “Senile Osteoporoses” OR “Bone Loss, Age-Related” OR “Age-Related Bone Loss” OR “Bone Loss, Age Related” OR “Age-Related Osteoporosis” OR “Age-Related Osteoporoses” OR “Osteoporoses, Age-Related”  #1 AND #2 | 5 |
| (3) Web of science | #1  TOPIC:  ((“Epimedium” or “Epimediums” or “Epimedium sagittatum” or “Epimedium sagittatums” or “sagittatum, Epimedium” or “sagittatums, Epimedium” or “Epimedium grandiflorum” or “Epimedium grandiflorums” or “grandiflorum, Epimedium” or “grandiflorums, Epimedium” or “Epimedii Folium”) )  Indexes=SCI-EXPANDED, SSCI, A&HCI, CPCI-S, CPCI-SSH, BKCI-S, BKCI-SSH, ESCI, CCR-EXPANDED, IC Timespan=All years  #2  TITLE:  ((“Osteoporosis” or “Osteoporosis, Senile” or “Osteoporoses, Senile” or “Senile Osteoporoses” or “Senile Osteoporosis” or “Osteoporosis, Age Related” or “Bone Loss, Age-Related” or “Age-Related Bone Loss” or “Age-Related Bone Losses” or “Bone Loss, Age Related” or “Bone Losses, Age-Related” or “Age-Related Osteoporosis” or “Age Related Osteoporosis” or “Age-Related Osteoporoses” or “Osteoporoses, Age-Related”) )  Indexes=SCI-EXPANDED, SSCI, A&HCI, CPCI-S, CPCI-SSH, BKCI-S, BKCI-SSH, ESCI, CCR-EXPANDED, IC Timespan=All years  #3  ALL FIELDS:  (randomized)  Indexes=SCI-EXPANDED, SSCI, A&HCI, CPCI-S, CPCI-SSH, BKCI-S, BKCI-SSH, ESCI, CCR-EXPANDED, IC Timespan=All years  #3 AND #2 AND #1  Indexes=SCI-EXPANDED, SSCI, A&HCI, CPCI-S, CPCI-SSH, BKCI-S, BKCI-SSH, ESCI, CCR-EXPANDED, IC Timespan=All years | 1 |
| (4) Google Scholar | (“Osteoporosis” OR “Osteoporoses, Senile” OR “Age-Related Bone Loss” OR “Osteoporoses”) AND (“Epimedium” OR “Epimedium sagittatum” OR “Epimedium grandiflorum” OR “Epimedii Folium”) AND (“randomized controlled trial”) | 258 |
| (5) WorldCat | #1  kw:“Epimedium” or “Epimediums” or “Epimedium sagittatum” or “Epimedium sagittatums” or “sagittatum, Epimedium” or “sagittatums, Epimedium” or “Epimedium grandiflorum” or “Epimedium grandiflorums” or “grandiflorum, Epimedium” or “grandiflorums, Epimedium” or “Epimedii Folium”  #2  ti:“Osteoporosis” or “Osteoporosis, Senile” or “Osteoporoses, Senile” or “Senile Osteoporoses” or “Senile Osteoporosis” or “Osteoporosis, Age Related” or “Bone Loss, Age-Related” or “Age-Related Bone Loss” or “Age-Related Bone Losses” or “Bone Loss, Age Related” or “Bone Losses, Age-Related” or “Age-Related Osteoporosis” or “Age Related Osteoporosis” or “Age-Related Osteoporoses” or “Osteoporoses, Age-Related”  #1 AND #2 | 0 |
| (6) Cochrane Library | #1  MeSH descriptor: [Epimedium] explode all trees  #2  (“Epimedium” or “Epimediums” or “Epimedium sagittatum” or “Epimedium sagittatums” or “sagittatum, Epimedium” or “sagittatums, Epimedium” or “Epimedium grandiflorum” or “Epimedium grandiflorums” or “grandiflorum, Epimedium” or “grandiflorums, Epimedium” or “Epimedii Folium”):ti,ab,kw  #3  #1 or #2  #4  MeSH descriptor: [Osteoporosis] explode all trees  #5  (Osteoporosis or “Osteoporosis, Senile” or “Osteoporoses, Senile” or “Senile Osteoporoses” or “Senile Osteoporosis” or “Osteoporosis, Age Related” or “Bone Loss, Age-Related” or Age-Related Bone Loss or Age-Related Bone Losses or “Bone Loss, Age Related” or “Bone Losses, Age-Related” or Age-Related Osteoporosis or Age Related Osteoporosis or Age-Related Osteoporoses or “Osteoporoses, Age-Related”):ti,ab,kw  #6  #4 or #5  #7  #3 And #6 | 12 |
| (7) Embase | ('epimedium':ti,ab,kw OR 'epimediums':ti,ab,kw OR 'epimedium sagittatum':ti,ab,kw OR 'epimedium sagittatums':ti,ab,kw OR 'sagittatum, epimedium':ti,ab,kw OR 'sagittatums, epimedium':ti,ab,kw OR 'epimedium grandiflorum':ti,ab,kw OR 'epimedium grandiflorums':ti,ab,kw OR 'grandiflorum, epimedium':ti,ab,kw OR 'grandiflorums, epimedium':ti,ab,kw OR 'epimedii folium':ti,ab,kw) AND ('osteoporosis':ti,ab,kw OR 'osteoporosis, senile':ti,ab,kw OR 'osteoporoses, senile':ti,ab,kw OR 'senile osteoporoses':ti,ab,kw OR 'senile osteoporosis':ti,ab,kw OR 'osteoporosis, age related':ti,ab,kw OR 'bone loss, age-related':ti,ab,kw OR 'age-related bone loss':ti,ab,kw OR 'age-related bone losses':ti,ab,kw OR 'bone loss, age related':ti,ab,kw OR 'bone losses, age-related':ti,ab,kw OR 'age-related osteoporosis':ti,ab,kw OR 'age related osteoporosis':ti,ab,kw OR 'age-related osteoporoses':ti,ab,kw OR 'osteoporoses, age-related':ti,ab,kw) AND 'randomized' | 19 |
| (8) SinoMed | #1  “淫羊藿”[关键词:智能] OR “仙灵脾”[常用字段:智能]  #2  “骨质疏松”[加权:扩展]  #3  “随机”[全部字段:智能]  (#1) AND (#2) AND (#3) | 22 |
| (9) China National Knowledge Infrastructure Database | #1 ((篇关摘=“淫羊藿”) OR (篇关摘=“仙灵脾”)  #2 (篇关摘=“骨质疏松”) OR (篇关摘=“骨质丢失, 年龄相关”)  #3 (全文=“随机”)  #1 AND #2 AND #3 | 539 |
| (10) Wan Fang database Search strategy | 题名或关键词:( “淫羊藿” or “仙灵脾” ) and 题名或关键词:(“骨质疏松” or “骨质丢失, 年龄相关”) and 全部:(“随机”) | 192 |
| (11) Chongqing VIP Chinese Science and Technology Periodical Database (VIP) | #1  题名或关键词=“淫羊藿” or “仙灵脾”  #2  题名或关键词=“骨质疏松” or “骨质丢失, 年龄相关”  #1 AND #2 | 291 |
| Overall |  | 1,346 |
